# Male and Female Mice Show Similar Fear Memory Performance Despite Hippocampal Immediate Early Gene Expression Differences During Encoding and Consolidation

**DOI:** 10.3390/cells15171523

**Published:** 2026-08-24

**Authors:** Katherine O. McDonald, Temmie Yu, Aditi Prabhu, Sara J. Aton

**Affiliations:** Department of Molecular, Cellular, and Developmental Biology, University of Michigan, Ann Arbor, MI 48109, USA; mckate@umich.edu (K.O.M.); temmieyu@umich.edu (T.Y.); aprabhu@umich.edu (A.P.)

**Keywords:** hippocampus, memory encoding, memory consolidation, memory retrieval, neural networks, immediate early genes

## Abstract

**Highlights:**

**What are the main findings?**

- Male and female mice perform similarly in a well-studied experimental memory task, contextual fear memory (CFM).
- In the absence of significant behavioral differences, male and female mice show differing hippocampal cFos expression patterns during CFM processing.

**What is the implication of the main finding**?

- Without comparing neural mechanisms of memory processing between males and females, our understanding of the neurobiological substrates of memory will remain limited and incomplete.
- The neurophysiological or genetic underpinnings of memory processing may differ substantially between males and females, even when memory recall measures are similar.

**Abstract:**

Accurate and efficient memory processing is essential for survival. A body of ongoing work in both human subjects and animal models suggests that memory processing may differ substantially between males and females. In mice, contextual fear memory (CFM) encoding, consolidation, and recall have been well studied, and the mouse hippocampus and amygdala have been implicated in these processes. The present pilot study addresses whether the activation of these brain regions differs substantially between male and female mice at each stage of CFM processing. We find that male and female mice show no differences in sleep behavior, which is essential for CFM consolidation, following single-trial contextual fear conditioning (CFC). We also find no significant differences in CFM recall performance between male and female mice. However, females show a trend for larger increases in CA1 cFos expression, relative to males, during CFM encoding. On the other hand, only males—but not females—show an apparent increase in cFos expression among dentate gyrus (DG) granule cells during CFM consolidation. Males also show a trend for a larger apparent reduction in cFos in CA1 and CA3 during CFM consolidation, relative to females. These preliminary findings highlight the idea that the neurobiological underpinnings of memory processing may differ between males and females, even when performance during recall is identical.

## 1. Introduction

Over the past 50 years, our understanding of biological processes underlying memory processing—an understanding essential for preserving healthy cognition—has advanced substantially. However, many of the cellular, molecular, and systems-level mechanisms of memory processing have been described in studies using only male animals [1]. This is surprising, as many studies have found substantial differences in memory processing mechanisms between males and females [2]. Closing this knowledge gap has important implications. For example, men and women differ in their rate of Alzheimer’s disease diagnosis across all age groups [3,4], and the genetics associated with cognitive resilience and susceptibility to dementia can also differ between males and females [5,6]. Understanding sex differences in the memory processing mechanisms may allow more tailored interventions for Alzheimer’s disease and other disorders characterized by disrupted cognition. The exclusion of female animals from studies addressing memory mechanisms severely limits our ability to extrapolate experimental findings to the human population.

Episodic memories—memories of events, associations, and contexts—are disrupted in many neurological disorders and rely heavily on hippocampal and neocortical regions for proper encoding, consolidation, and recall [7,8,9,10]. Contextual fear memory (CFM) is among the best-studied episodic-like memory used in laboratory mice, where it can be encoded in a single learning trial, referred to as contextual fear conditioning (CFC). CFC itself activates the amygdala and hippocampus [11,12], and CFM is consolidated in the hours following CFC via a mechanism that relies on dorsal hippocampal neural activity [13], gene transcription [14,15], and protein translation [16,17,18]. While the process of CFM consolidation is not fully understood, post-CFC sleep appears to play an important role via its effects on hippocampal and neocortical activity patterns [19,20,21,22] and its activation of critical intracellular pathways [15,16,23,24,25,26]. More recent work has shown that CFM recall evokes reactivation of the same neurons activated during CFC—including populations of so-called “engram” neurons in the hippocampus and amygdala [15,27].

Historically, most research on CFM has been carried out using only male animals, excluding females, with the aim of reducing potential variability in behavior or physiology across the estrus cycle [1,28]. A few recent studies have begun to test sex as a biological variable affecting CFM processing. Some of these comparative studies have shown that male rodents show either greater contextual freezing responses or more specific CFM responses than females (i.e., with less context generalization) during CFM recall [29,30,31]. However, others identified either no behavioral differences between males and females (and even similar behavioral variability between males and cycling females [28,32]) or greater freezing responses in females [29]. The mechanistic underpinnings of these putative sex-based CFM differences are also unclear. For example, CFM consolidation is dependent on post-CFC sleep [15,19,23,25,33], and differences in sleep architecture between male and female rodents have been reported [34,35,36]. It is unknown whether these sleep architecture differences could translate to differences in CFM consolidation between males and females. Intriguingly, even in studies where CFM recall performance appears equal between males and females, the cellular pathways required for memory processing may vary with sex [37,38,39]. In the context of these varying findings, it remains unclear whether the activity of circuits engaged by CFM processing also differs between males and females [2,40]. Our present pilot study reveals potential sex differences in the activity of hippocampal and amygdalar structures during CFM encoding, consolidation, and recall, in the absence of significant behavioral differences. Our data highlight the need for further investigation of different brain regions’ contribution to memory processing in males vs. females.

## 2. Methods

### 2.1. Mouse Husbandry, Handling, and Experimental Groups

Prior to each experiment, C57BL6/J male (*n* = 17) and female (*n* = 18) mice (Jackson; 3–6 months of age) were maintained on a 12 h light/12 h dark cycle (lights on at 9 am) under controlled temperature and relative humidity conditions (22 ± 2 °C; 60–75%). Mice were group-housed in standard filter-top caging with age-matched littermates (no more than 5 to a cage), provided beneficial enrichment (Enviro-Dry nesting packets), and allowed *ad lib* access to food and water. Beginning on the first day of habituation to experimental handling, each mouse was single-housed with additional enrichment (cotton nestlets) to decrease effects of cage mate interactions on post-learning sleep and remained single-housed for the duration of behavioral experiments (4–5 days total for all groups). All animal husbandry and experimental procedures were approved by the University of Michigan Institutional Animal Care and Use Committee (IACUC). Because behavioral outcomes in mice can vary with the sex of the experimenter [41], all handling procedures were carried out by a female experimenter.

**Figure 1:**
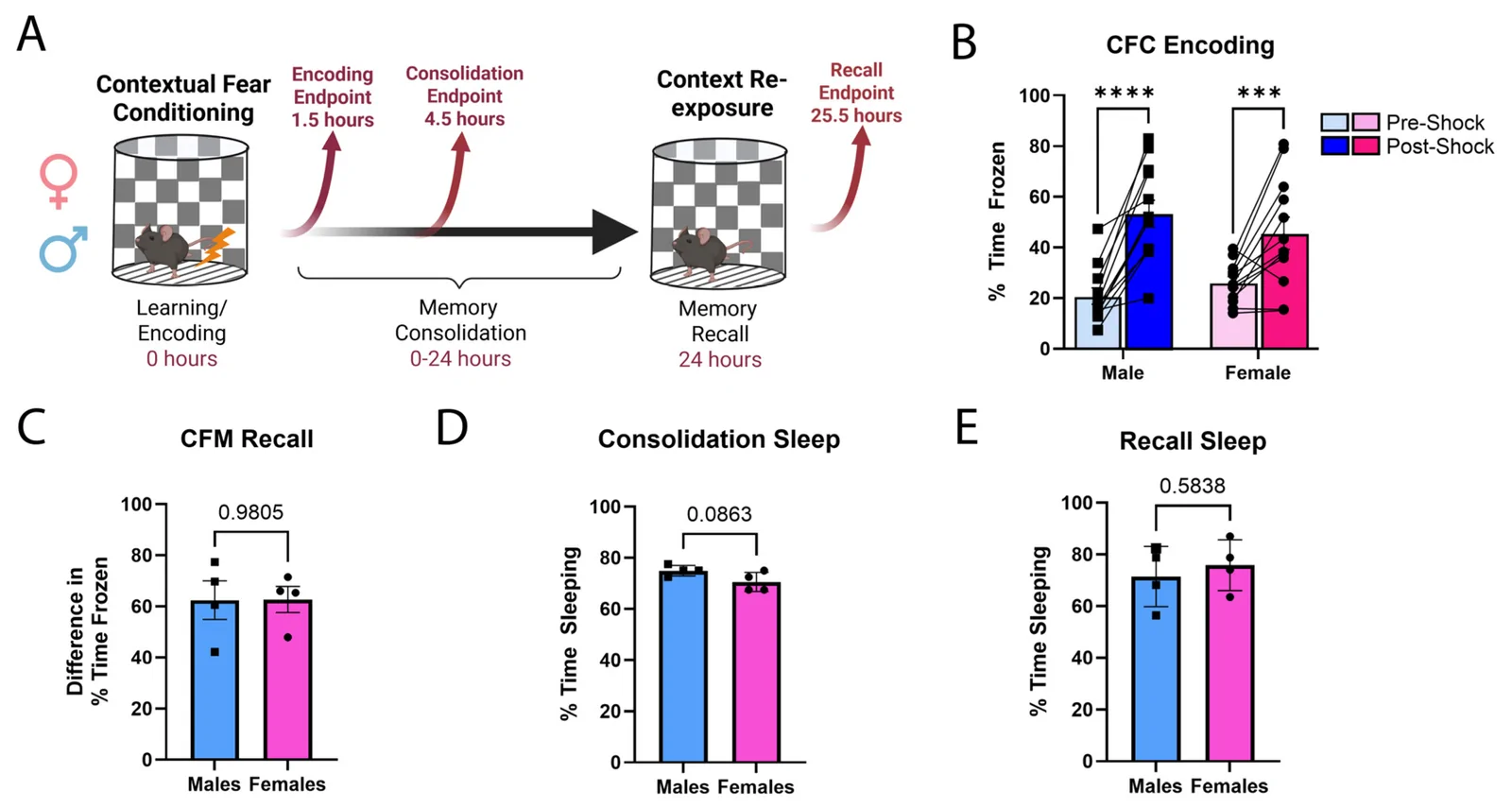
**Male and female mice show similar behavioral responses to CFC, post-CFC sleep, and CFM recall.** (**A**) Male and female experimental mice underwent single-trial CFC, then were euthanized for immunohistochemical assessment of neural activity patterns during CFM encoding, consolidation, or recall. (**B**) During single-trial CFC, both male and female mice showed significantly greater freezing post-shock compared to pre-shock (*n* = 12 male, 12 female, two-way ANOVA with uncorrected Fisher’s LSD males post-shock vs. pre-shock, **** indicates *p* < 0.0001, females post- to pre-shock, *** indicates *p* = 0.0008, other comparisons *p* > 0.05, main effect of sex F [1, 22] = 0.082, *p* = 0.78, η^2^ = 0.0014; main effect of memory stage F [1, 22] = 55.69, *p* < 0.0001, η^2^ = 0.42; sex × memory stage interaction effect F [1, 22] = 3.9, *p* = 0.061, η^2^ = 0.029). (**C**) Male and female mice showed similar CFM recall—measured as % change in context-specific freezing—during testing 24 h following CFC (*n* = 4 males, 4 females, male vs. female *p* = 0.98, unpaired Student’s *t*-test, t = 0.026, df = 6, R^2^ = 0.00011). (**D**) Male and female mice from the CFM consolidation group did not significantly differ in % time asleep over the 4.5 h immediately following CFC (males mean ± SEM = 75% ± 1.021%, female average mean ± SEM = 70.63% ± 1.88%, *n* = 4 males 4 females, male vs. female *p* = 0.086, unpaired Student’s *t*-test t = 2.05, df = 6, R^2^ = 0.42). (**E**) Male and female mice from the CFM recall group also did not significantly differ in % time spent sleeping over the first 6 h following CFC (males mean ± SEM = 71.47% ± 5.83%, female average mean ± SEM = 75.88% ± 4.91%, *n* = 4 males, 4 females, male vs. female *p* = 0.58, unpaired Student’s *t*-test t = 0.58, df = 6, R^2^ = 0.053). All values indicate mean ± SEM.

Prior to each experiment, all mice were handled daily for 3 days (4 min/day) for habituation to experimenter handling procedures. Mice were assigned to experimental timepoint groups for measuring activity-driven cFos expression during CFM encoding (*n* = 4 male, 4 female), consolidation (*n* = 4 male, 4 female), or recall (*n* = 4 male, 4 female) (Figure 1A). A group of age-matched control mice (*n* = 5 male, 6 female) underwent the same housing and handling procedures but did not undergo CFC. To minimize batch effects, control and experimental mice of both sexes underwent behavioral procedures simultaneously, and the number of batches (i.e., cohorts of mice representing all experimental groups) was limited. Individual male or female mice born in the same litter were assigned to groups randomly, distributing littermates between experimental groups so that: (1) no two mice in a single group came from the same litter and (2) there was an even age distribution across groups. The sex of all mice was verified during daily handling. Experimental data for a single male mouse assigned to the consolidation control group was removed from subsequent analysis due to being incorrectly sexed at the time of weaning.

### 2.2. Contextual Fear Conditioning (CFC) and Contextual Fear Memory (CFM) Testing

After 3 days of handling, experimental mice underwent single-trial CFC, as previously described [15]. At lights on (Zeitgeber Time [ZT] 0), each mouse was placed in a novel cylindrical plexiglass conditioning chamber (22 cm diameter × 25 cm high) with a black-and-white checkerboard pattern on the interior walls, a metal grid floor for foot shock delivery (Med Associates), and an associated scent cue (lemon Lysol). Mice were allowed to explore this environmental context for 2 min 28 s before receiving a 2 s, 0.75 mA foot shock, followed by an additional 30 s in the chamber, and were then returned to their home cage. Behavior during CFC and CFM recall was video monitored. Freezing behavior during both recordings was quantified using an automated analysis algorithm in EthoVision software (v.17; Noldus Information Technology, Wageningen, The Netherlands). For each mouse, CFM-associated freezing behavior was expressed as (% of time spent freezing during the 5 min recall session) − (% of time freezing in the 1 min 28 s period prior to CFC foot shock) to account for individual differences in baseline freezing, as done previously [19,20,21].

Timepoints for euthanasia and brain tissue harvest were selected based on prior studies indicating that cFos protein expression peaks roughly 1.5 h following experimentally induced neuronal activity [42,43,44,45]. At 1.5 h after CFC (a timepoint at which cFos protein expression would be expected to reflect neuronal activity associated with CFM encoding), mice in the encoding experimental group (*n* = 4 male, 4 female) and 3 time-matched, no-CFC control mice (*n* = 1 male, 2 female) were euthanized with an i.p. injection of pentobarbital (Euthasol; Butler Schein, Dublin, OH, USA) prior to transcardial perfusion with 15 mL of ice-cold phosphate-buffered saline (PBS), followed by 15 mL of ice-cold 4% paraformaldehyde. Mice in the consolidation experimental group (*n* = 4 male, 4 female) were euthanized and perfused 4.5 h after CFC (to quantify neural cFos expression associated with active CFM consolidation) [15,25], along with 4 time-matched control mice (*n* = 2 male, 2 female). The 4.5 h post-CFC timepoint corresponded to cFos protein expression driven by brain activity after 3 h of post-learning sleep, a time period during which cellular pathways associated with memory consolidation mechanisms are upregulated in the dorsal hippocampus [25,26,46]. The next day at lights on, mice in the recall experimental group (*n* = 4 male, 4 female) were re-exposed to the CFC context for 5 min for assessment of CFM recall (i.e., freezing behavior) and were then returned to their home cage. These mice and 4 no-CFC control animals (*n* = 2 male, 2 female) were euthanized and perfused 1.5 h after CFM recall to capture cFos expression (a proxy measure for neuronal activity) associated with recall. Because circadian rhythms of cFos expression and various activity-regulated cellular pathways have been reported in the rodent hippocampus [47,48], we compared cFos expression levels in each of the measured brain structures in control mice as a function of ZT at the time of tissue harvest (Supplementary Figure S2).

### 2.3. Sleep Monitoring

All mice were allowed *ad lib* sleep in their home cages, with visual sleep monitoring starting either immediately after CFC (for experimental animals) or at lights on (for control animals). Sleep behavior was identified visually based upon mice becoming quiescent, nesting, and assuming typical sleep postures (crouched within the nest with head down, motionless outside of regular respiratory movements). This non-invasive method for identifying sleep behavior is comparable to polysomnographic recording [49] and can be carried out without prior surgical procedures and minimal prior single housing. Each mouse in the consolidation and recall groups was observed continuously over the first 4.5–6 h following CFC (i.e., 4.5 h between CFC and sacrifice for mice in the consolidation group, and the first 6 h post-CFC for the recall group). The first few hours following CFC is a window of time during which uninterrupted sleep is critical for successful CFM consolidation [15,19,23,25,33]. Mice were determined to be either asleep or awake once every 5 min, in real time, by a single researcher blinded to the experimental condition, across this 4.5–6 h time window. For each mouse, sleep behavior was quantified as a % of the total observation period.

### 2.4. Estrus Cycle Monitoring

Because vaginal smear collection for cytological assessment can cause distress and disrupt cognition in female mice [50], assessment of the estrus cycle stage in females was carried out during tissue harvest by imaging the anogenital area for visual assessment of the exterior vaginal canal, using established methods [51]. Distributions of estrus cycle phases for females in each experimental group are shown in Supplementary Table S1. The estrus cycle could not be confidently determined in two female animals, and thus their cycle stage is not reported.

### 2.5. Immunohistochemistry and Image Analysis

Immediately following perfusion, brains were extracted, incubated in 4% paraformaldehyde at 4 °C for 24 h, then transferred to ice-cold phosphate-buffered saline (PBS) for coronal sectioning (80 µm thickness) using a vibratome (Leica VT1200s; Leica, Teaneck, NJ, USA). Coronal sections approximately −1.58 to −2.18 mm from bregma were selected for immunostaining. Sections were blocked in PBS with 1% Triton X-100 and 5% normal donkey serum for 24 h at 4 °C, incubated for 4 days at 4 °C in primary antibody (rabbit anti cFos 1:750 [Abcam, Waltham, MA, USA; ab190-289]), washed in 0.2% Triton X-100 in PBS, and incubated for 2 days at 4 °C with secondary antibody (DyLight 633 donkey anti rabbit igG 1:800 [Novus Biologicals, Centennial, CO, USA; NBP1-75638]). Antibody dilutions and incubation periods were based on prior studies of neuronal activation in the mouse hippocampus following CFC [15,52]. After immunostaining, brain sections were washed in PBS, incubated at room temperature in DAPI (1:500) for 5 min to stain cell nuclei, re-washed, and mounted and coverslipped with ProLong Gold antifade reagent (ThermoFisher, Waltham, MA, USA; P36930). All tissue samples were prepared simultaneously. Immunolabeled brain sections were imaged using identical settings using a Leica SP8 laser scanning confocal microscope at 40× magnification. For each brain region quantified, 4–5 serial sections were imaged for each animal. Quantifications for each of these sections were averaged to obtain a single numeric value for each region.

Brain structures within acquired images were mapped in reference to the Allen Brain Reference Atlas, using QuPath image analysis software (v.0.5.1; https://qupath.github.io/, accessed on 20 August 2026). For all hippocampal subregions (CA1–CA3 and dentate gyrus [DG]), analysis of cFos expression was constrained to the principal cell body layers. To ensure that CA1–CA3 measurements were fully contained within the appropriate subregions (i.e., where anatomical borders between these regions are not clearly defined), analysis was constrained to the center of each region (approximately 100 µm from the borders of neighboring regions of interest, based on border locations referenced in the Allan Brain Reference Atlas), avoiding areas close to the border with other CA structures. Individual cells were detected within each brain region using DAPI fluorescence. For all brain regions analyzed, QuPath software was used for intensity threshold-based detection of cFos+ cells; automated detection of cFos+ cells was curated by an experimenter blinded to the sex and experimental conditions of each mouse. For all ROIs quantified, a DAPI+-identified nucleus was considered cFos+ if the average pixel intensity within its boundaries was >175 (on a scale from 0 to 255). This threshold was chosen based on the mean fluorescence intensity outside of DAPI+ areas across all ROIs of all subregions measures. cFos+ ratios were calculated as the number of cFos+ cells over the total number of DAPI+ cells per region.

For all image analyses, values for experimental mice (male or female encoding, consolidation, or recall) were compared to a group of control mice (*n* = 5 male, 6 female) that did not undergo CFC and were pooled across timepoints. Sample collection timepoints did not affect cFos expression levels among control mice.

### 2.6. Statistical Analysis

**Figure 2:**
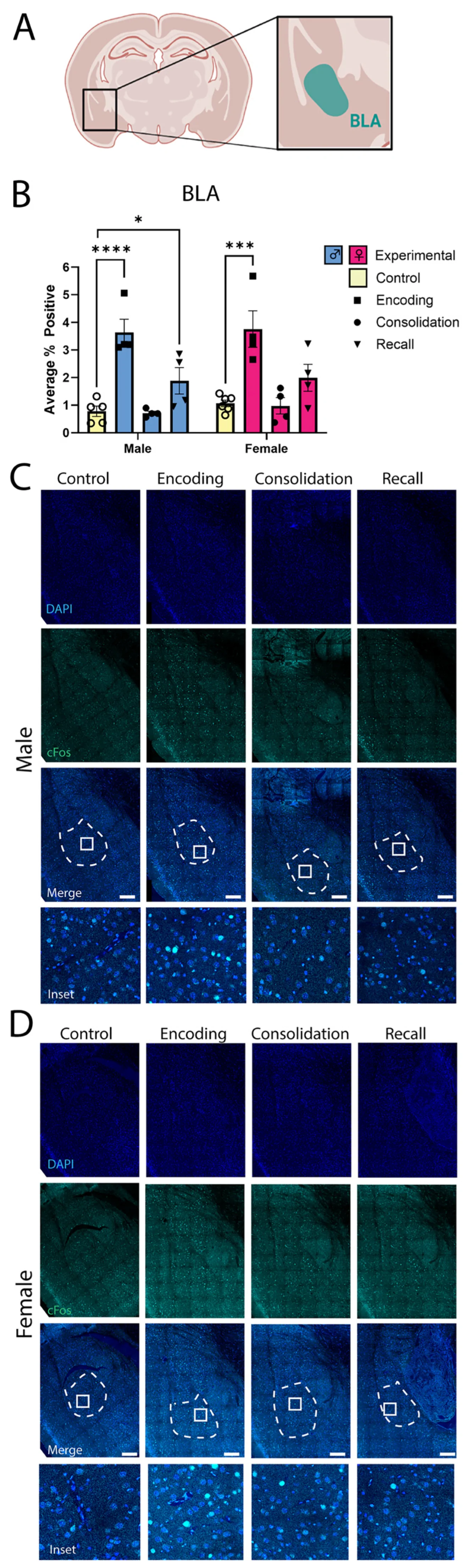
**Basolateral amygdala (BLA) neuron activation during CFM encoding is similar between male and female mice.** (**A**) BLA was immunolabeled to compare the density of cFos+ neurons across each stage of CFM processing (encoding, consolidation, and recall) in male vs. female mice. (**B**) In both males and females, the proportion of cFos+ BLA neurons was significantly increased during CFM encoding (two-way ANOVA with Dunnett’s *post hoc* test, males encoding vs. control, **** indicates *p* < 0.0001, female encoding vs. control, *** indicates *p* = 0.0007). Additionally, males—but not females—showed significantly greater BLA activity during recall compared to controls, although there was no significant main effect of sex or sex x memory stage interaction (male recall vs. control, * indicates *p* = 0.018; all other pairwise comparisons *p* > 0.15; main effect of sex F [1, 27] = 0.55, *p* = 0.46, η^2^ = 0.00043; main effect of memory stage F [3, 27] = 25.46, *p* < 0.0001, η^2^ = 0.025; sex × memory stage interaction effect F [3, 27] = 0.032, *p* = 0.99, η^2^ = 0.00044). All values indicate mean ± SEM. (**C**,**D**) Representative images of immunolabeled (**C**) male and (**D**) female BLA structures with DAPI+ and cFos+ immunolabeling. Outlined regions (dashed lines) indicate ROIs used for analysis. Boxes indicate location of insets of higher magnification, in bottom rows. Scale bars = 250 μm.

**Figure 3:**
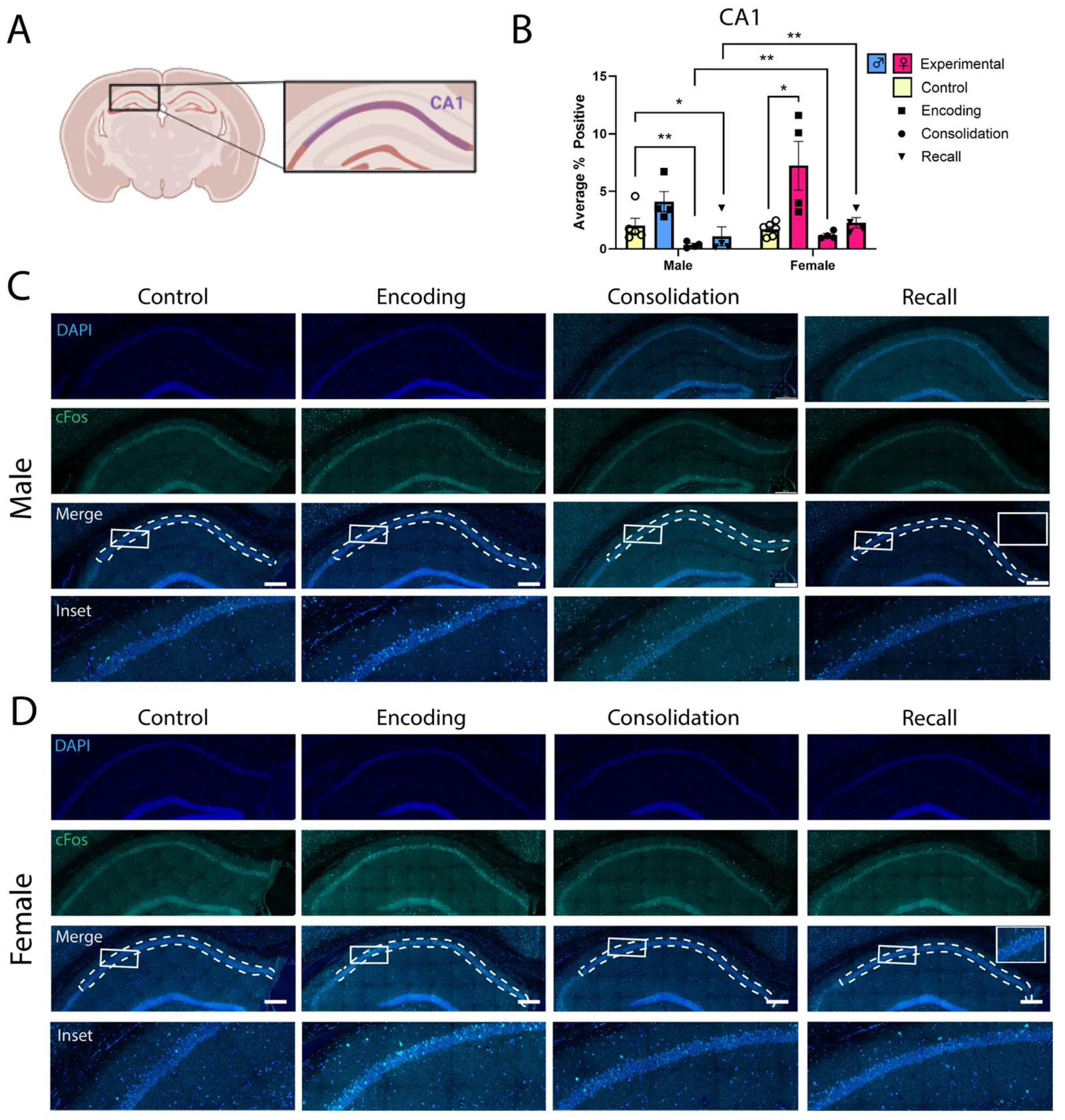
**CA1 pyramidal neuron activity differs between male and female mice across CFM processing stages.** (**A**) The proportion of cFos+ neurons in the dorsal CA1 pyramidal cell body layer was quantified for male and female mice in each group. (**B**) Female mice—but not male mice—had a significantly greater proportion of cFos+ CA1 neurons after CFM encoding compared to controls (two-way ANOVA with Dunnett’s *post hoc* test, female encoding vs. control, ** indicates *p* = 0.016). Additionally, male mice—but not female mice—showed a reduction in cFos+ CA1 neuron proportions during both CFM consolidation and recall compared to control conditions (two-way ANOVA with Dunnett’s *post hoc* test, male consolidation vs. control, ** indicates *p* = 0.0014, male vs. female consolidation, ** indicates *p* = 0.0063; male recall vs. control, * indicates *p* = 0.027, male vs. female recall, ** indicates *p* = 0.0046; all other comparisons *p* > 0. 22), with significant main effects of both sex and memory stage (*n* = 5 male, 6 female control; 4 male, 4 female encoding; 4 male, 4 female consolidation; 4 male, 4 female recall; main effect of sex F [1, 27] = 12.75, *p* = 0.0014, η^2^ = 0.14; main effect of memory stage F [3, 27] = 14.16, *p* < 0.0001, η^2^ = 0.48; sex × memory stage interaction effect F [3, 27] = 2.83, *p* = 0.057, η^2^ = 0.096). (**C**,**D**) Representative CA1 images with DAPI and cFos immunolabeling for (**C**) male and (**D**) female mice in each group. Outlined regions (dashed lines) indicate ROIs used for analysis. Boxes indicate location of insets of higher magnification, in bottom rows. Values indicate mean ± SEM. Scale bars = 250 μm.

**Figure 4:**
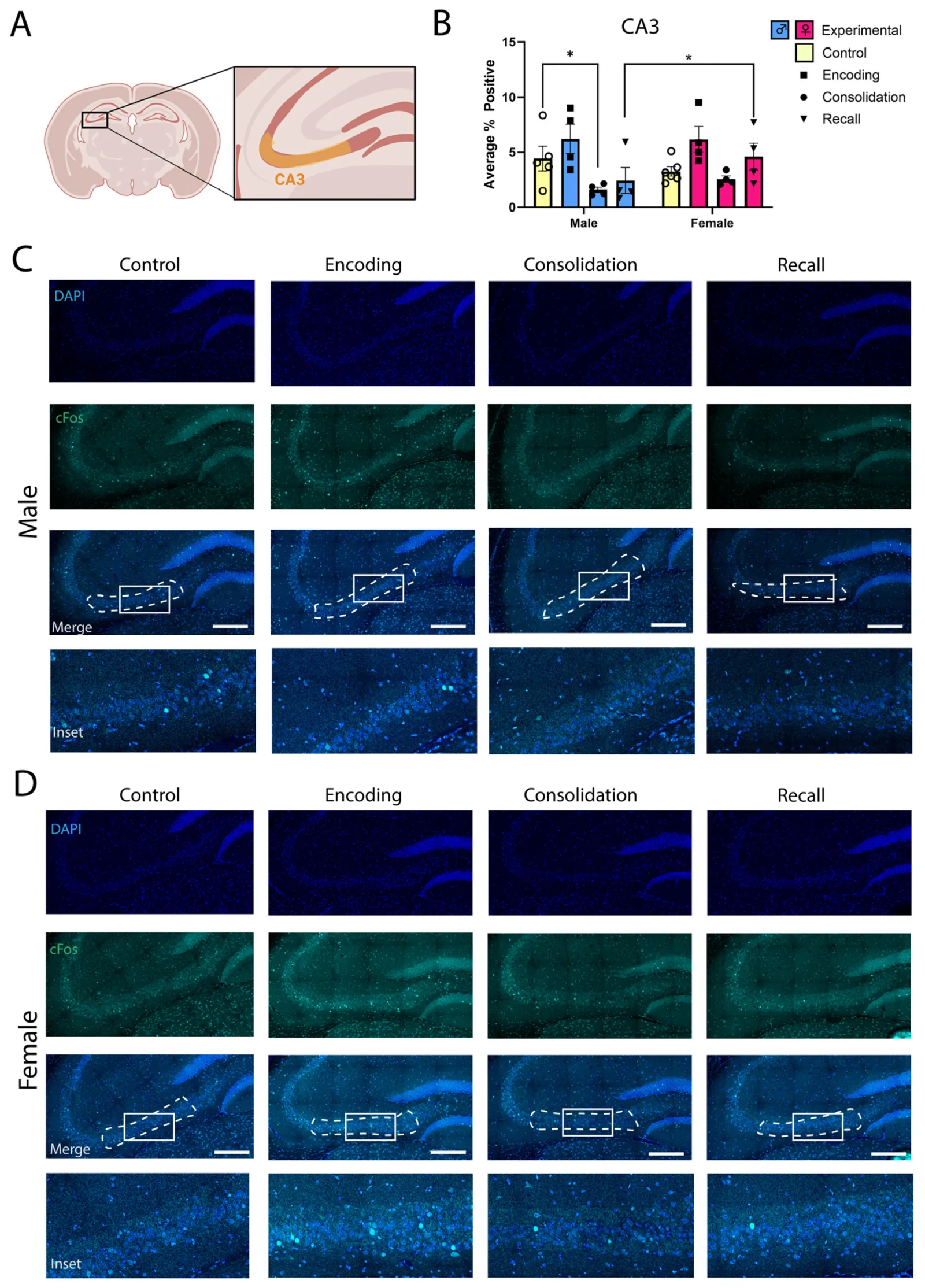
**CA3 pyramidal neuron activity is suppressed during CFM consolidation in male mice**. (**A**) The proportion of cFos+ dorsal CA3 pyramidal neurons was compared between male and female mice at each CFM processing stage. (**B**) Male mice had significantly lower cFos+ neuron density during consolidation compared to controls (two-way ANOVA with Dunnett’s *post hoc* test, male consolidation vs. control, * indicates *p* = 0.024). Other groups were not significantly different from controls (all other comparisons, *p* > 0.07), with a significant overall main effect of memory stage (*n* = 5 male, 6 female control; 4 male, 4 female encoding; 4 male, 4 female consolidation; 4 male, 4 female recall; main effect of sex: F [1, 27] = 2.9, *p* = 0.099, η^2^ = 0.052; main effect of memory stage: F [3, 27] = 7.05, *p* = 0.0012, η^2^ = 0.38; sex × memory stage interaction: F [3, 27] = 2.031, *p* = 0.13; η^2^ = 0.11). Female mice showed greater cFos+ neuron proportions during recall compared to males (Dunnett’s *post hoc* test, * indicates *p* < 0.025). (**C**,**D**) Representative CA3 images from (**C**) male and (**D**) female mice in each group. Outlined regions (dashed lines) indicate ROIs used for analysis. Boxes indicate location of insets of higher magnification, in bottom rows. Values indicate mean ± SEM. Scale bars = 250 μm.

**Figure 5:**
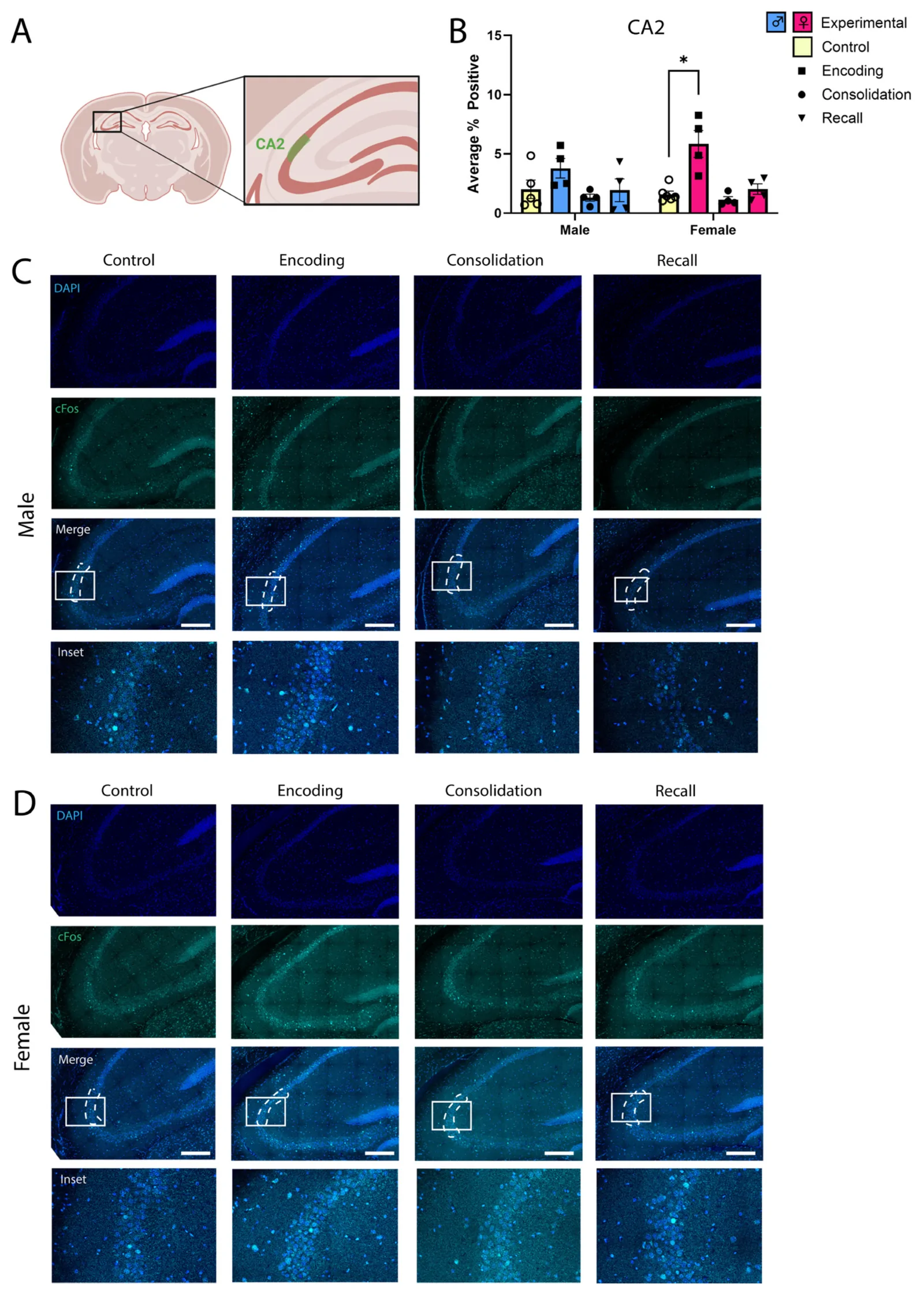
**CA2 pyramidal neuron activity is significantly increased during CFM encoding in female mice.** (**A**) cFos+ neuron density was quantified in the pyramidal cell body layer of dorsal CA2. (**B**) Female mice had significantly greater CA2 cFos+ neuron density during encoding compared to controls (two-way ANOVA with Dunnett’s *post hoc* test female encoding vs. controls, * indicates *p* = 0.0153), while the same was not true for the male cohort (all other pairwise comparisons *p* > 0.17), with a significant overall main effect of memory stage (*n* = 5 male, 6 female control; 4 male, 4 female encoding; 4 male, 4 female consolidation; 4 male, 4 female recall; main effect of sex F [1, 27] = 0.9250, *p* = 0.6847, η^2^ = 0.019; main effect of memory stage F [3, 27] = 6.754, *p* = 0.0015, η^2^ = 0.41; sex × memory stage interaction effect F [3, 27] = 0.50, *p* = 0.69; η^2^ = 0.030. (**C**,**D**) Representative CA2 images from (**C**) male and (**D**) female mice in each group. Outlined regions (dashed lines) indicate ROIs used for analysis. Boxes indicate location of insets of higher magnification, in bottom rows. Values indicate mean ± SEM. Scale bars = 250 μm.

**Figure 6:**
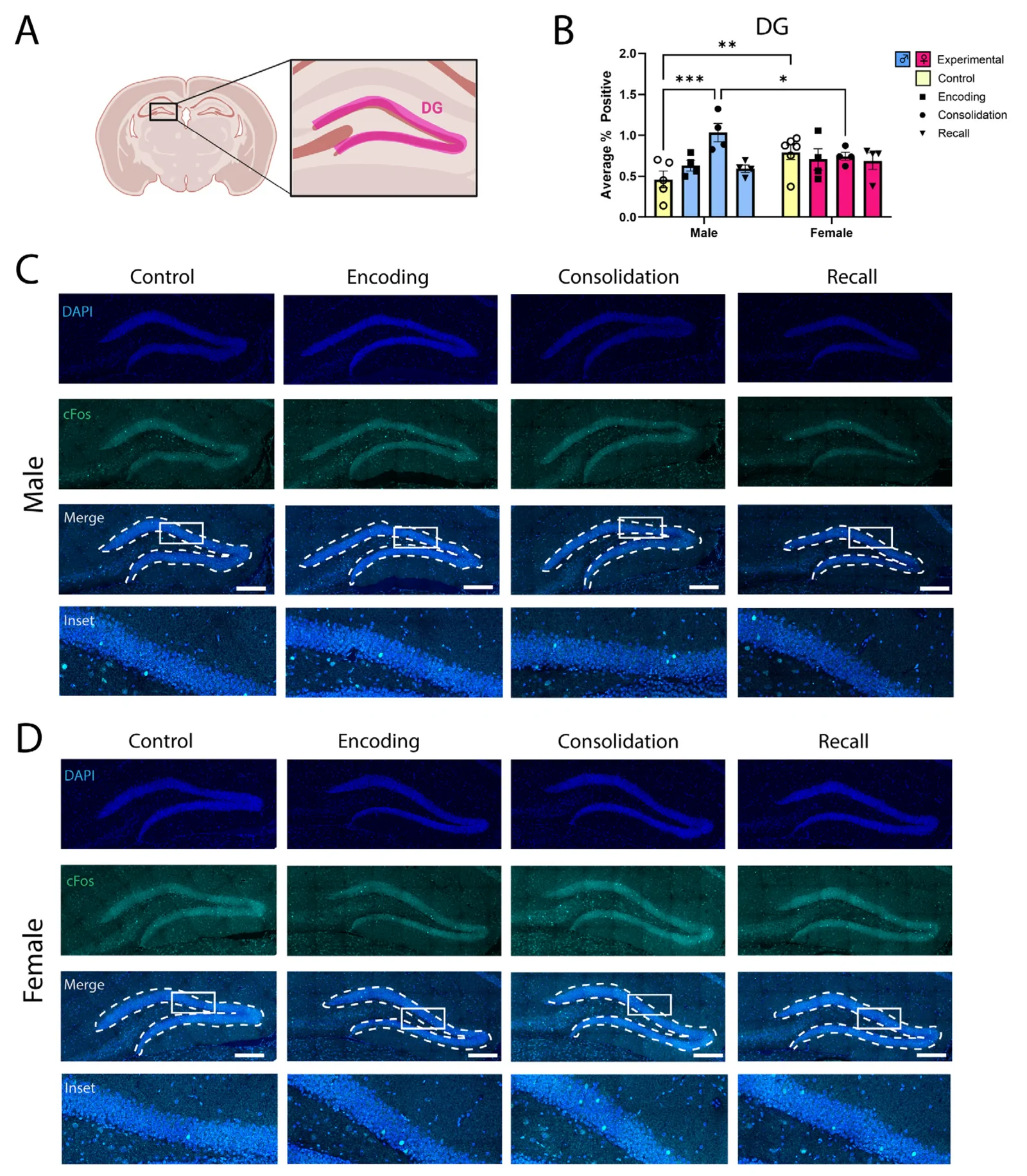
**DG granule cell activity increases during CFM consolidation in male mice only.** (**A**) cFos expression was quantified in the granule cell body layers of dorsal hippocampal DG (inferior and superior blade). (**B**) Male mice—but not female mice—showed significantly greater cFos+ neuron density during CFM consolidation compared to controls (two-way ANOVA with Dunnett’s *post hoc* test male consolidation vs. control, *** indicates *p* = 0.0009, male consolidation vs. female consolidation, * indicates *p* = 0.046, male control vs. female control, ** indicates *p* = 0.0095), with a significant sex × memory stage interaction effect (*n* = 5 male, 6 female control; 4 male, 4 female encoding; 4 male, 4 female consolidation; 4 male, 4 female recall; main effect of sex F [1, 27] = 0.62, *p* = 0.44, η^2^ = 0.013; main effect of memory stage: 3.34, *p* = 0.034, η^2^ = 0.20; sex × memory stage interaction: F [3, 27] = 3.88, *p* = 0.02; η^2^ = 0.24). (**C**,**D**) Representative DG images from (**C**) male and (**D**) female mice in each group. Outlined regions (dashed lines) indicate ROIs used for analysis. Boxes indicate location of insets of higher magnification, in bottom rows. Values indicate mean ± SEM. Scale bars = 250 μm.

For all analyses comparing two groups (e.g., Figure 1C–E), simple unpaired *t*-tests were used for statistical analyses. For all comparisons of more than two groups or multiple timepoints (Figure 1B, Figure 2, Figure 3, Figure 4, Figure 5 and Figure 6), two-way ANOVA was performed. All two-way ANOVA analyses compared experimental group means (e.g., encoding, consolidation, and recall) of both sexes (male and female) to male and female control group means. The normality of residuals for all analyses were assessed with the Shapiro–Wilk test and passed normality assessment (alpha = 0.05). Datasets that were not normally distributed (Figure 3B and Figure 4B) were log-transformed for analysis with two-way ANOVA. All transformed datasets passed the Shapiro–Wilk normality test. Heteroscedasticity was assessed using the Spearman’s rank correlation test for two-way ANOVAs or using the *F*-test for unpaired *t*-tests. Datasets that did not pass the heteroscedasticity test were log-transformed and reported statistics run on transformed data (Figure 2B,D, Figure 4B and Figure S2B,D,F). All *t*-tests passed the *F*-test to compare variances. All transformed datasets are displayed in their raw form, with appropriate statistical markers for clarity. For analyses that compared data of the same set of mice over multiple timepoints (Figure 1B), a two-way ANOVA with uncorrected Fisher’s LSD was performed. For analyses that compared multiple brain regions over multiple timepoints (Supplementary Figure S2B,D,F), a two-way ANOVA with Šidák’s multiple comparisons test was performed. For all other two-way ANOVAs, Dunnett’s *post hoc* test was performed to correct for multiple comparisons. Effect sizes are reported as R^2^ for all unpaired *t*-tests, and η^2^ for all two-way ANOVAs. All data were analyzed using GraphPad Prism 11.0.0. For correlations of cFos expression among different subregions (Supplementary Figures S2 and S3), Pearson correlation coefficients (r) were calculated using raw or transformed values as described above, as appropriate. Correlations with ZT and estrus cycle stage with expression levels were calculated using ZT numeric values and with cycle stages converted to integer values (proestrus = 1, estrus = 2, diestrus = 3, metestrus = 4).

## 3. Results

### 3.1. Male and Female Mice Show Comparable CFM Recall Performance and Post-CFC Sleep Behavior

We first compared males’ and females’ learning-related behavior during single-trial CFC, as well as during subsequent CFM recall (Figure 1A and Figure S1). Males and females did not differ in the amount of time spent in freezing behavior during CFC itself (i.e., during CFM encoding) (Figure 1B; males pre-shock mean ± SEM = 20.52 ± 3.17%, males post-shock mean ± SEM = 54.21 ± 5.43%, females pre-shock mean ± SEM = 26.01 ± 2.3%, females post-shock mean ± SEM = 45.59 ± 6.53%, main effect of sex: F [1, 22] = 0.082, *p* = 0.78, main effect of memory stage: F [1, 22] = 55.69, *p* < 0.0001, sex × memory stage interaction: F [1, 22] = 3.9, *p* = 0.061). Both male and female mice showed increased freezing behavior upon return to the CFM context (Figure 1C; 62.5 ± 7.6% and 62.7 ± 5.1% [mean ± SEM] increases in freezing for males and females, respectively), and CFM recall performance did not differ between males and females (*p* = 0.98, unpaired *t*-test;) (Figure 1C and Figure S1). Because episodic memory consolidation is highly dependent on sleep [15,19,23,24,25,33,53], mice from consolidation and recall groups underwent visual scoring of sleep behavior over the first 4.5 h or 6 h, respectively, following CFC. There were no significant differences between male and female mice with respect to sleep behavior during this time window (consolidation group males and females: 75.0 ± 1.02% and 70.63 ± 1.88% [mean ± SEM] of 4.5 h and 6 h post-CFC monitoring period spent asleep, respectively, *p* = 0.086 unpaired *t*-test; recall group males and females 71.47 ± 5.83% and 75.88 ± 4.91% [mean ± SEM] time spent asleep, respectively, *p* = 0.58 unpaired *t*-test) (Figure 1C,D).

### 3.2. Amygdalar Activity Increases in Response to Fear Learning in Both Males and Females

Fear memory processing has been shown to activate neurons in amygdalar structures, including the basolateral amygdala (BLA) [27,54]. To test whether sex differences in amygdalar activation were present in the absence of a behavioral difference in CFM recall, we quantified the proportion of amygdalar neurons that were cFos+ during CFM encoding, consolidation, and recall stages (Figure 2A). We compared the proportion of amygdalar cells expressing cFos in males vs. females at each stage of CFM processing, identifying changes in mice undergoing CFC vs. home cage control mice. Male and female mice showed a similar degree of BLA activation immediately following CFC, with a significant increase in the cFos+ cell proportion during encoding compared to controls, which returned to control levels during CFM consolidation (Figure 2B–D). During CFM recall, the proportion of cFos+ BLA cells tended to again be elevated relative to controls, but this reached statistical significance in males only.

### 3.3. Hippocampal CA1–CA3 Activity Patterns Differ Between Males and Females Across CFM Encoding, Consolidation, and Recall

Neuronal activity in dorsal hippocampal regions CA1 and CA3 is necessary for successful CFM processing [13]. To test for potential sex differences in the dorsal hippocampus during CFM processing, we next compared the proportion of CA1 neurons in the pyramidal cell body layer that were cFos+ in males and females across encoding, consolidation, and recall (Figure 3A–D). Our data indicate that in CA1 of females—but not males—activity-driven cFos expression during encoding is significantly elevated compared to control animals (two-way ANOVA with Dunnett’s *post hoc* test, male encoding vs. control *p* = 0.22, female encoding vs. control *p* = 0.016; Figure 3B). Interestingly, the proportion of cFos+ CA1 neurons was significantly *decreased* during both CFM consolidation (vs. control *p* = 0.0014) and recall (vs. control *p* = 0.027) in male mice—but not female mice—compared to controls. The proportion of cFos+ CA1 neurons was higher during both consolidation and recall in female mice compared to male mice (Dunnett’s *post hoc* test, male vs. female, *p* = 0.0063 and *p* = 0.0046 for consolidation and recall, respectively).

In contrast to CA1, CA3 (Figure 4A–D) did not show a significant increase in cFos+ neuron density during CFM encoding in either male or female mice. Female mice—but not male mice—showed a trend for an increased proportion of cFos+ CA3 neurons during encoding (two-way ANOVA with Dunnett’s *post hoc* test, male encoding vs. control *p* = 0.49, female encoding vs. control *p* = 0.15). In contrast, male mice—but not female mice—showed a significant *reduction* in cFos+ neuron density in CA3 during memory consolidation (Dunnett’s *post hoc* test, male consolidation vs. control *p* = 0.023; Figure 4B) and a similar trend during recall (*p* = 0.074). As was true in CA1, the proportion of cFos+ CA3 neurons during recall was higher in males than females (Dunnett’s *post hoc* test, *p* = 0.025).

While the roles of CA1 and CA3 in CFM processing have been well studied, contributions of adjacent CA2 are less clear [55]. Similarly to our findings in CA1, CA2 (Figure 5A–D) had significantly more cFos+ cells during CFM encoding compared to controls in female—but not male—mice (two-way ANOVA with Dunnett’s *post hoc* test, *p* = 0.015). Unlike CA1 or CA3, no significant changes in CA2 cFos expression were observed in either sex during the consolidation or recall of CFM (Figure 5B).

### 3.4. Dentate Gyrus (DG) Activity Is Increased During Active CFM Consolidation in Male Mice Only

The hippocampal DG is another structure engaged by CFC encoding and recall, which more recently has been implicated in CFM consolidation [15,46,52]. However, aside from a recent report of greater DG activity during CFC in males vs. females [29], it is largely unknown whether sex differences in CFM processing-associated activity exist within this region. While in hippocampal CA1-CA3, cFos expression was highest during CFC and no increase was observed during CFM consolidation (Figure 3, Figure 4 and Figure 5), no increase in DG cFos expression was observed during CFC in either males or females (Figure 6A–C). In contrast, during CFM consolidation, the proportion of cFos+ DG neurons increased in male mice (Figure 6D; two-way ANOVA with Dunnett’s *post hoc* test, *p* = 0.0009 for male consolidation vs. controls), with a significant sex × memory stage interaction effect (F [3, 27] = 3.88, *p* = 0.02) and a significant difference between males and females at this memory processing stage (*p* = 0.046, Dunnett’s *post hoc* test). Surprisingly, in females, there were no significant differences in DG activity levels relative to controls at any stage of CFM processing (Figure 6D). Importantly, CFM consolidation is highly dependent on post-CFC sleep [15,19,25], and DG “engram neuron” reactivation in male mice is sleep dependent [15]. However, no significant differences in post-CFC sleep behavior were observed between male and female experimental mice (Figure 1C). Together, these data suggest that DG activation patterns during CFM consolidation differ between male and female mice, and that these differences may not be attributable to differences in post-CFC sleep.

## 4. Discussion

The findings from this pilot study add to a growing body of data suggesting that neural activation patterns differ between male and female mice in some—but not all—brain regions during CFM processing. Regions in which we observed significant or trending sex differences, and/or sex × memory stage interaction effects, in cFos expression included dorsal hippocampal areas CA1, CA3, and DG (Figure 3, Figure 4 and Figure 6). BLA cFos expression was similar between male and female mice, with both showing significant increases in cFos during CFM encoding, and a similar trend for increases during recall (Figure 2B). In nearly all brain regions quantified, we found a significant main effect of the memory processing stage (i.e., encoding, consolidation, or recall), but in BLA and CA2, there were neither significant nor trending effects of sex nor sex × memory stage interaction effects.

There is a growing body of research that suggests there may be sex differences in CFM processing in mice [29,31]. Recent data suggest biological sex and sex hormones can affect the extent of CFM context generalization and CFM extinction behaviors [29,56,57]. Moreover, males and females differ with respect to how specific experimental conditions—e.g., single housing or the sex of the experimenter—affect their freezing scores [41,58]. However, there are also several studies finding no observable sex differences in CFM recall performance, despite the cellular and molecular mechanisms required for CFM differing between males and females [37,38,39]. Similarly, in our present study, no apparent differences in contextual freezing were observed between males and females during either training or testing (Figure 1); yet, differences in hippocampal activity patterns during encoding and consolidation were present (Figure 3, Figure 4 and Figure 6).

One interesting trend we observed was the comparatively high activation of both CA1 and CA2 neurons during CFM encoding in females, relative to males (Figure 3 and Figure 5). While CA1 has been well studied with respect to contextual memory processing [13,19,20,21], much less is known about the contribution of adjacent CA2. While CA2 has synaptic connections with CA1, CA3, DG, and the entorhinal cortex [59,60], the region long had a contested role in memory processing due to its unique resistance to synaptic plasticity [61]. More recently, CA2 has been implicated in social memory processing [62,63,64], and our present findings contribute to a growing body of evidence suggesting it may play a role in processing other memory types [55,65]. However, unlike CA1, there is no statistical trend for greater activity in CA2 during CFM encoding in females vs. males. Thus, our data suggest that while CFC leads to activation of both CA2 and CA1, the extent of neural activation in CA1 may be affected, to some degree, by sex.

The most surprising findings from the present study are the apparent differences in dorsal hippocampal activity between males and female mice during CFM consolidation. During the consolidation timepoint examined here, males showed significantly higher DG cFos expression (Figure 6) and significantly lower CA1 and CA3 cFos expression (Figure 3 and Figure 4), compared both to controls and to female mice. This suggests that male mice may have reduced activity in CA1 and CA3 and increased DG activity during CFM consolidation. All three regions are implicated in episodic memory formation and recall [13,15,27,52,66,67,68], and recent work from our lab and others suggests that they also play essential roles in CFM consolidation [15,46]. Our prior work—using primarily male mice—has suggested that the success of CFM consolidation is tightly correlated with expression patterns of cFos, Arc, and activity-regulated post-translational events among DG granule cells [15,46,49]. It is unclear whether, or how, elevated activity of DG granule cells is mechanistically related to the low level of CA1 and CA3 pyramidal neuron activity during CFM consolidation in males. However, recent data from our lab indicate that patterns of DG interneuron activation can substantially influence activity in both CA structures [52]. These consolidation-associated cFos expression changes were not observed to the same extent in female mice. We hope that results from the present pilot study will be hypothesis generating with respect to the nature of these sex differences. Since CFM consolidation is clearly unimpaired in female mice, these data suggest that: (1) other circuit-level mechanisms may be involved in the consolidation process in females; (2) other activity-regulated pathways (i.e., besides cFos) may come into play within hippocampal subregions, in ways that differ between males and females [69,70]; and/or (3) differences in encoding between males and females (with encoding evoking stronger activation of CA regions in females) may lead to a stronger memory trace without the need for high levels of DG activity in the hours following CFC.

Interestingly, some studies find that males outperform females on context discrimination tasks [29]. Thus, one possibility is that greater DG network activity during consolidation [15], and/or reduced activity of pyramidal cells in CA1 and CA3, contributes to this difference by improving pattern separation in males [71,72]. This hypothetical function, linking sex differences in either activity-driven intracellular mechanisms within DG or DG circuit activity to specific differences in cognition, warrants further investigation.

There are several important limitations to data interpretation for the present pilot study. First is the relatively small sample sizes for experimental groups. These preliminary experiments were not powered to detect group differences with small effect sizes. Indeed, for statistically significant group differences reported in these studies, the effect sizes were quite large (i.e., η^2^ > 0.2). For this reason, negative results from these studies should be interpreted with extreme caution. Second, while some (but not all) studies have reported changes in CFM as a function of the estrus cycle in female rodents [58,73,74,75], it is unclear whether, or how, those changes are linked to brain activity patterns during CFM processing. Our present data do not suggest that the variability of either CFM performance or cFos expression patterns in female mice differs from that of male mice. While we found no significant correlation between the estrus cycle stage and cFos expression levels in any brain region (Supplementary Figure S2), these experiments were not statistically powered to test for differences in cFos expression across the estrus cycle (Supplementary Table S1). Moreover, as the cycle stage was determined *post hoc* and not controlled for prior to experiments (Supplementary Table S1), it is still plausible that cycling hormones contribute to some observed sex differences in hippocampal brain activity. Thus, future studies will be needed to test whether cycling hormones affect the hippocampal network’s activity during CFM processing, and how this might relate to behavioral outcomes. It is also worth noting that while cFos is widely used as an immunohistochemical indicator of prior neuronal activity, it is only one of several such indicators, which include cFos, Arc, Zif268, Npas4, and numerous post-translational protein modifications. These various indicators can differentially respond to activity within different cell populations [46,70,76,77]. Critically, because the expression of other neuronal activity markers may differ between males and females [69], we cannot rule out any sex differences to activity-regulated protein abundance or post-translation modifications. In other words, measurement of cFos alone is unlikely to provide a holistic characterization of neuronal or network activity patterns. Lastly, while we did not find significant differences in visually scored sleep time between male and female mice (Figure 1D,E), differences in sleep architecture—e.g., differences in the relative proportion of NREM vs. REM sleep, or state-specific activity patterns like sleep spindles—may still be present. Future studies using a combination of in vivo calcium imaging and polysomnographic recording may determine whether the observed sex differences in cFos expression during CFM consolidation are driven by more subtle sex differences in post-CFC sleep architecture or neuronal activity.

It is worth noting parallels between the findings of this study and recent findings from studies on human subjects. Sex differences have been observed in healthy adults with respect to recall of emotionally valanced episodic and autobiographical memories [78,79,80], as well as in memory performance in male vs. female patients with mild cognitive impairment or post-traumatic stress disorder [81,82]. In many human studies—but not all—differences in performance have been linked to changes in BOLD signal in the hippocampus, amygdala, and/or neocortical structures during memory encoding and/or recall [78,79,80]. While neither human fMRI data nor the cFos expression patterns measured here are direct measures of neuronal firing activity (and are not directly comparable to each other) [83], these findings contribute to the idea that cognitive processing in men and women may differentially engage canonical “memory” circuitry. As was the case with our present findings, performance on some tasks, such as spatial navigation, is equivalent between men and women, despite apparent sex differences in connectivity between the brain structures they engage [84]. One important consideration for interpreting our current findings is that men and women have been shown to have different developmental trajectories for wiring connections within the limbic system—both during adolescence [85] and in adulthood [86]. Men and women also differ with respect to age-related degeneration and metabolic changes within these structures [87]. Future studies will be needed to assess how these sex differences over the lifespan affect the dynamic functioning of brain circuitry in the context of healthy cognition. However, data such as the present findings of this pilot study suggest that sex differences in spatial and temporal circuit dynamics could reflect different neurobiological processes that achieve the same cognitive outcome.

## 5. Conclusions

In the absence of fear memory performance differences, male and female mice show differing hippocampal cFos expression patterns during fear memory processing. The present findings of this pilot study suggest that sex differences in spatial and temporal circuit dynamics could reflect different neurobiological processes that achieve the same cognitive outcome.

## Data Availability

Data used in these studies will be made available upon reasonable request.

## Supplementary Materials

The following supporting information can be downloaded at: https://www.mdpi.com/article/10.3390/cells15171523/s1, Figure S1. Locomotor activity patterns during CFC and CFM recall. Figure S2. Regional cFos expression in control mice is not determined by time of day, or estrus cycle stage in females. Figure S3. Correlation of cFos expression across brain structures in experimental mice, as a function of memory processing stage. Table S1. Estrus cycle stages of female mice in each experimental group.
