# Supplementary material for: Male and Female Mice Show Similar Fear Memory Performance Despite Hippocampal Immediate Early Gene Expression Differences During Encoding and Consolidation
Source: Cells. 2026 Aug 24;15(17):1523. doi: 10.3390/cells15171523 (PMC13565567; doi:10.3390/cells15171523)
Supplement: Supplementary file 1 [file cells-15-01523-s001.zip › cells-4458884-supplementary.pdf]

Supplementary Materials:

Table S1. Estrus cycle stages of female mice in each experimental group. Estrus cycle stage for 16/18 female mice was assessed visually prior to euthanasia. Stage could not be determined in 2/18 females.

	Proestrus (*n* = 1)	Estrus (*n* =7)	Metestrus (*n* =5)	Diestrus (*n* =3)
**Control**	0	2	2	2
**Encoding**	0	2	0	1
**Consolidation**	1	1	1	0
**Recall**	0	2	2	0

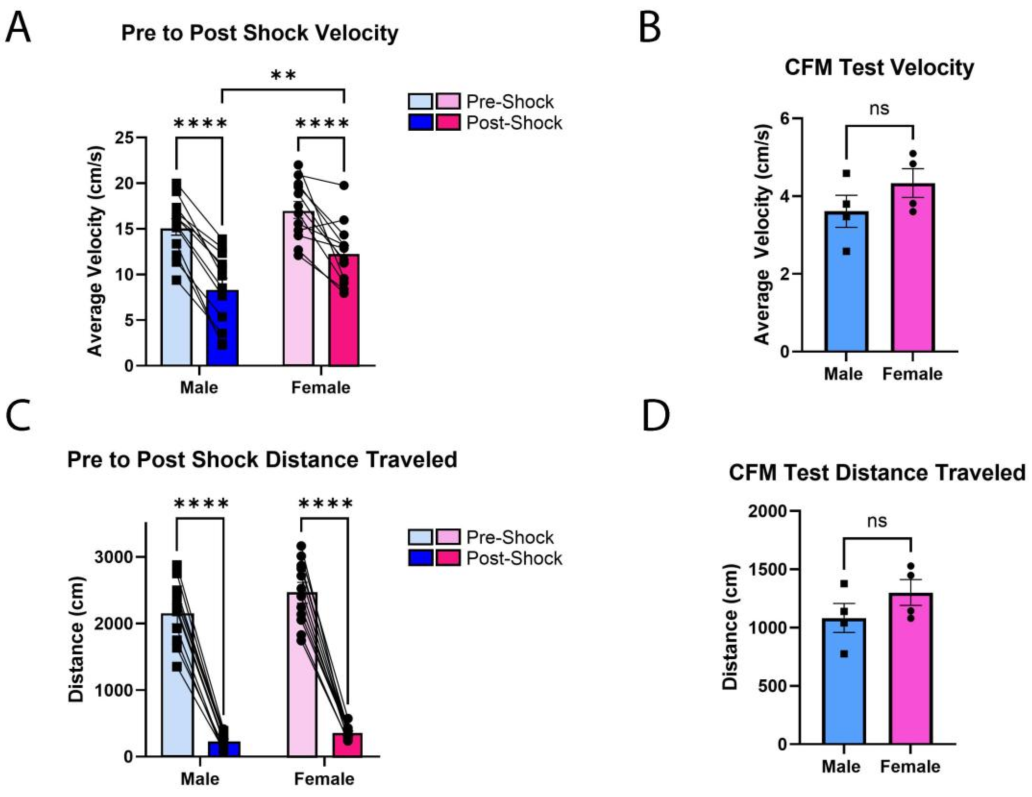

Figure S1. Locomotor activity patterns during CFC and CFM recall. A) The average velocity of mice within the testing chamber pre- and post-shock during CFC training was measured in cm/s and compared between males and females. Both male and female mice showed significant decreases in velocity following shock administration, consistent with increased post-shock freezing behavior (uncorrected Fisher's LSD male pre-shock vs. post-shock, **** indicates $p < 0.0001$, female pre-shock vs. post shock, **** indicates $p < 0.0001$). However, the post-shock velocity of female mice was greater than that of male mice (post-shock male vs. female, ** indicates $p = 0.0096$, all other comparisons $p > 0.05$) ($n = 12$ males, 12 females; main effect of sex: $F [1, 22] = 4.982$, $p = 0.0361$, $\eta^2 = 0.094$;

main effect of memory stage: F [1, 22] = 87.83, $p < 0.0001$, $\eta^2 = 0.38$; sex × memory stage interaction: F [1, 22] = 2.58, $p = 0.12$, $\eta^2 = 0.12$). **B)** The average velocity of male and female mice during CFM recall was similar (unpaired Student's t test $p > 0.05$, $n = 4$ males, 4 females, t = 1.31, df = 6, $R^2 = 0.22$). **C)** The total distance traveled pre-and post-shock during CFC was measured in cm and compared across sexes. Both male and female mice showed significant decreases in distance traveled following shock administration (two-way ANOVA with uncorrected Fisher's LSD male pre-shock vs. post-shock, **** indicates $p < 0.0001$, female pre-shock vs. post-shock, **** indicates $p < 0.0001$), and there were no significant differences between male and female mice (all other comparisons p > 0.05) ($n = 12$ males, 12 females; main effect of sex: F [1, 22] =2.93, $p = 0.10$, $\eta^2 = 0.0079$; main effect of memory stage: F [1, 22] = 631.6, $p < 0.0001$, $\eta^2 = 0.90$; sex × memory stage interaction: F [1, 22] = 1.04, $p = 0.32$, $\eta^2 = 0.0015$). **C)** The total distance traveled of mice within the testing chamber during CFM recall was similar between male and female mice (unpaired Student's t test $p > 0.05$, $n = 4$ males, 4 females, t = 1.31, df = 6, $R^2 = 0.22$).

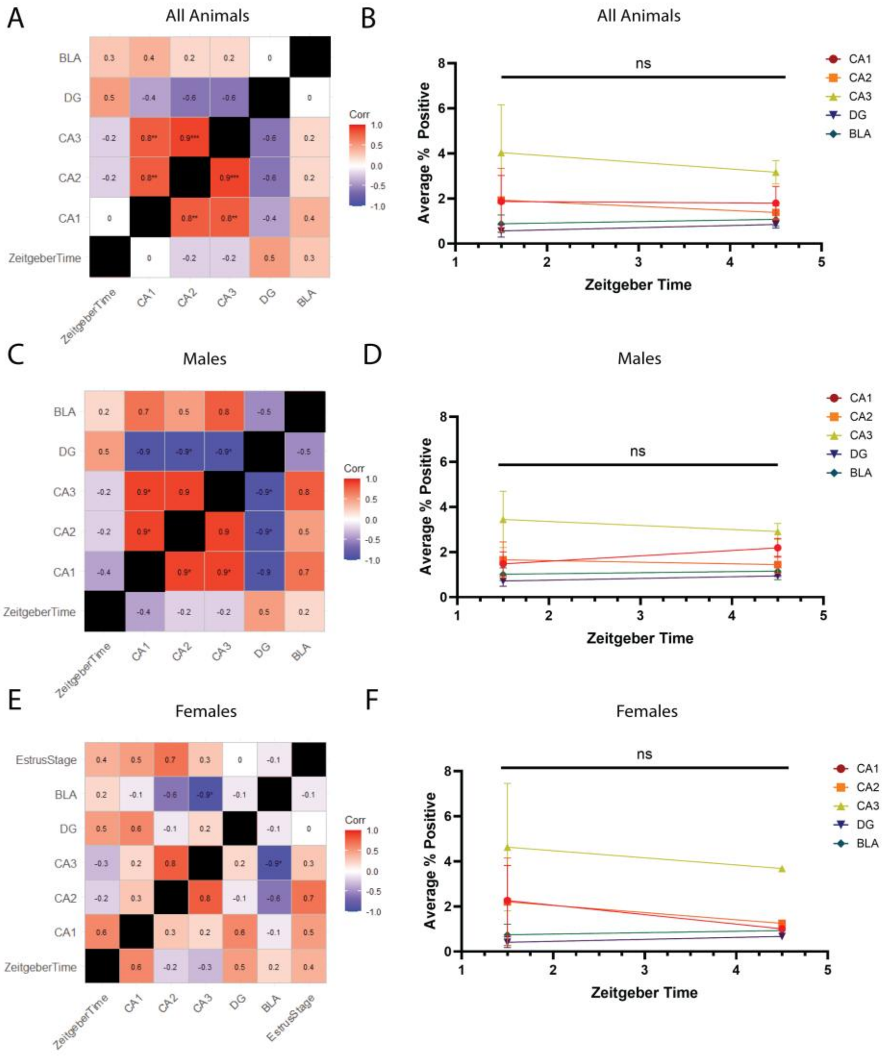

Figure S2. Regional cFos expression in control mice is not determined by time of day, or estrus cycle stage in females. A, C, E) In **A)** all control mice and **C)** male control mice, cFos expression was positively correlated among

CA1-3 subregions, with DG expression levels tending toward anticorrelation with values in CA1-3 (numeric values indicate Pearsons r; ***, **, * for $p < 0.001$, 0.01, 0.05 respectively). These relationships were not present in **E)** female control mice. Estrus cycle and Zeitgeber time (ZT) at tissue harvest were not significantly correlated to cFos levels in any brain region measured in control mice. **B, D, F)** Mean ± SD for % cFos+ neurons identified in each brain structure of control mice, as a function of ZT at tissue harvest. No significant effect of time of day on expression was found for any brain region. Two way ANOVA n = 5 male, 6 female control. For all control mice **(B)**, main effect of time point F [1, 45] = 0.518, $p = 0.476$, $\eta^2 = 0.00416$; main effect of brain subregion F[4, 45] = 12.59, $p < 0.0001$, $\eta^2 = 0.41$ time point x brain region interaction effect F [4, 45] = 0.70, $p = 0.60$; $\eta^2 = 0.0223$. **(D),** main effect of time point F [1, 15] = 0.00079, $p = 0.98$, $\eta^2 = 1.7\text{e-}005$; main effect of brain subregion F[4, 15] = 3.36, $p = 0.029$, $\eta^2 = 0.32$, time point x brain region interaction effect F [4, 15] = 0.40, $p = 0.80$; $\eta^2 = 0.035$. For female control mice (**F**), main effect of time point F [1, 20] = 1.08, $p = 0.31$, $\eta^2 = 0.013$; main effect of brain subregion F[4, 20] = 11.93, $p < 0.0001$, $\eta^2 = 0.58$, time point x brain region interaction effect F [4, 20] = 0.68, $p = 0.61$; $\eta^2 = 0.033$.)

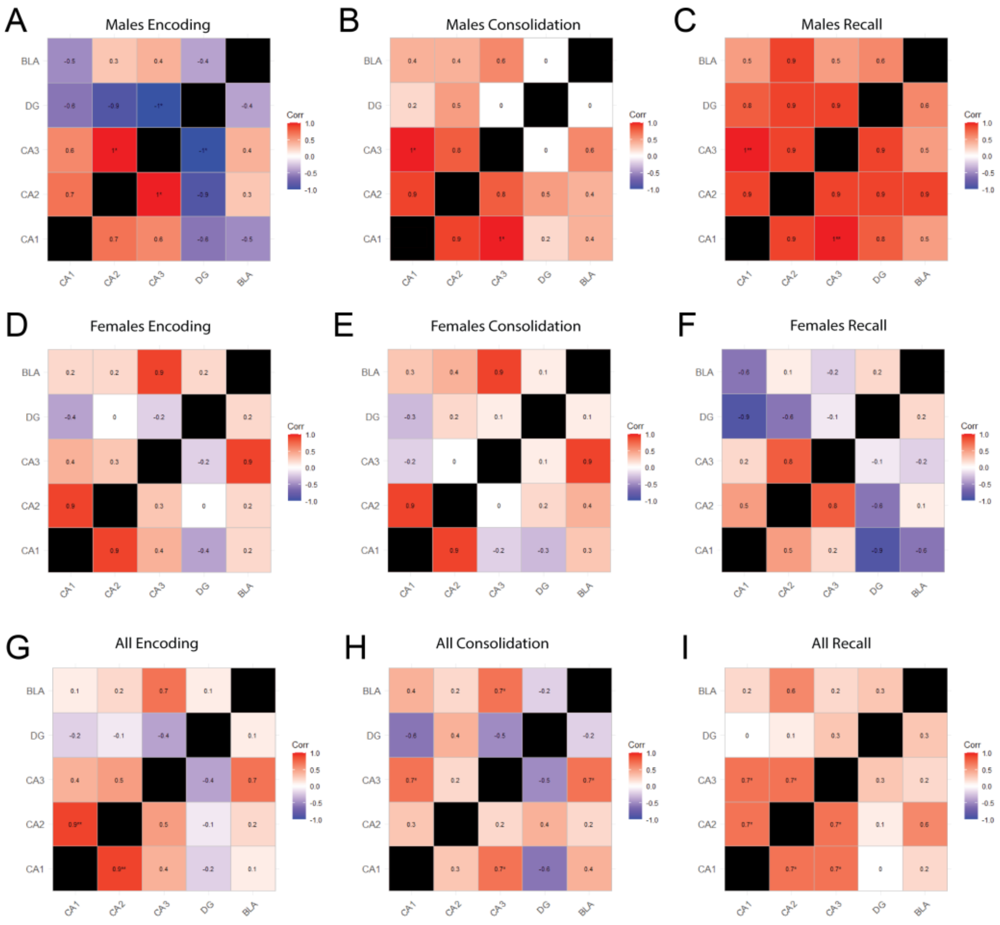

Supplementary Figure S3. Correlation of cFos expression across brain structures in experimental mice, as a function of memory processing stage. A-

C) In male experimental mice, (**A**) cFos expression was positively correlated between CA3 and CA2 and anticorrelated between CA3 and DG during encoding. During (**B**) consolidation and (**C**) recall, cFos expression in the CA3 and CA1 subregions was positively correlated. In female experimental mice, (**D-F**) no significant correlations between subregions were observed at any memory stage. When comparing values from all experimental mice (**G-I**), cFos expression in CA1 and CA2 were positively correlated during encoding. During (**H**) consolidation, cFos expression between CA3 and CA1, and between CA3 and BLA, was positively correlated. During (**I**) recall, cFos expression was positively correlated between CA1 and CA2, and betweenCA1 and CA3 (numeric values indicate Pearsons r; ***, **, * for $p < 0.001$, 0.01, 0.05 respectively).
